# Bait Effects in Sampling Coral Reef Fish Assemblages with Stereo-BRUVs

**DOI:** 10.1371/journal.pone.0041538

**Published:** 2012-07-27

**Authors:** Stacey R. Dorman, Euan S. Harvey, Stephen J. Newman

**Affiliations:** 1 School of Plant Biology and the UWA Oceans Institute (M470), University of Western Australia, Perth, Western Australia, Australia; 2 Western Australian Fisheries and Marine Research Laboratories, Department of Fisheries, Government of Western Australia, Perth, Australia; Biodiversity Insitute of Ontario - University of Guelph, Canada

## Abstract

Baited underwater video techniques are increasingly being utilised for assessing and monitoring demersal fishes because they are: 1) non extractive, 2) can be used to sample across multiple habitats and depths, 3) are cost effective, 4) sample a broader range of species than many other techniques, 5) and with greater statistical power. However, an examination of the literature demonstrates that a range of different bait types are being used. The use of different types of bait can create an additional source of variability in sampling programs. Coral reef fish assemblages at the Houtman Abrolhos Islands, Western Australia, were sampled using baited remote underwater stereo-video systems. One-hour stereo-video recordings were collected for four different bait treatments (pilchards, cat food, falafel mix and no bait (control)) from sites inside and outside a targeted fishery closure (TFC). In total, 5209 individuals from 132 fish species belonging to 41 families were recorded. There were significant differences in the fish assemblage structure and composition between baited and non-baited treatments (P<0.001), while no difference was observed with species richness. Samples baited with cat food and pilchards contained similar ingredients and were found to record similar components of the fish assemblage. There were no significant differences in the fish assemblages in areas open or closed to fishing, regardless of the bait used. Investigation of five targeted species indicated that the response to different types of bait was species-specific. For example, the relative abundance of *Pagrus auratus* was found to increase in areas protected from fishing, but only in samples baited with pilchards and cat food. The results indicate that the use of bait in conjunction with stereo-BRUVs is advantageous. On balance, the use of pilchards as a standardised bait for stereo-BRUVs deployments is justified for use along the mid-west coast of Western Australia.

## Introduction

Fish assemblages have been recognised as sensitive indicators of habitat degradation, ecosystem productivity and overall environmental change [1]. When establishing a study to assess changes in fish assemblage structure, it is important to understand how the utilised methodology influences the results. Baited remote underwater video systems (BRUVs) and their stereo-video counterparts (stereo-BRUVs) have been proposed as a novel, standardised, non-extractive methodology for estimating the relative abundance and diversity of demersal fishes, with the additional capability of compiling species-specific length data when stereo-video pairs are used [2]–[4]. When bait is used with a video camera deployment, the rate at which both the number of species and the number of individuals are sampled increases, reducing problems associated with low fish counts per sample [5]. Stereo-BRUV systems not only record species attracted to the bait, but also species that are present in the field of view by chance, attracted to the stereo-BRUVs structure, or to the behaviour of other fishes [2]. Both BRUVs and stereo-BRUVs have been demonstrated to be robust sampling tools for investigating spatial and temporal patterns in reef fish assemblages [2], [6]–[11]. In addition, the benefits and potential of the technique, such as their repeatability and cost effectiveness have been outlined in several comparative studies [3], [12]–[16]. However, the use of bait as an attractant in BRUVs studies continues to raise questions regarding bias and selectivity [2].

Potential biases associated with different types of bait create an additional source of variability in spatial and temporal sampling programs that could confound their ability to detect changes or differences [17]. Spatial or temporal changes in fish assemblages detected by BRUVs or stereo-BRUVs could potentially be due to changes in the type of bait used and the ability of the bait to sample representatively. Consequently, it has been recognised that the type, quantity and delivery of bait should be standardised [2]. The use of different bait types has been found to influence the abundance and species composition of fishes caught in commercial fish traps [18]–[20] and on long-lines [21]–[24]. Bait functions by releasing chemical stimuli (usually water-soluble proteins) into the surrounding water column, which is dispersed by the prevailing currents [18], [25]. The physical characteristics of bait, such as bait persistence and moisture content, govern soak time, dispersal area and the persistence of the bait plume [19], [26]. Bait must also be economically viable, environmentally sustainable and must not introduce pests or disease [27]–[29].

In commercial fish traps, fish abundance is increased by the use of oily, soft-fleshed baits such as pilchards (*Sardinops sagax*) as opposed to white-fleshed baits [18], [19], [30]. Consequently, pilchard and mixed pilchard combinations have been the only bait utilised in Australian and New Zealand BRUVs and stereo-BRUVs deployments [2], [4], [7], [8], [10], [14], [15], [31]–[39]. Given the widespread and increasing use of BRUVs and stereo-BRUVs [40], further research is needed to investigate the effects of bait. Factors such as the type and quantity of bait, the area covered by the resulting bait plume, the soak time and species-specific behavioural effects all influence the performance of baited video techniques [2], [3], [10], [13]. Wraith [41] investigated the effect of different bait types on the composition of a fish assemblage sampled by BRUVs. BRUVs baited with pilchards were found to attract a greater number of individuals and species than BRUVs baited with either urchin or abalone.

**Figure 1:**
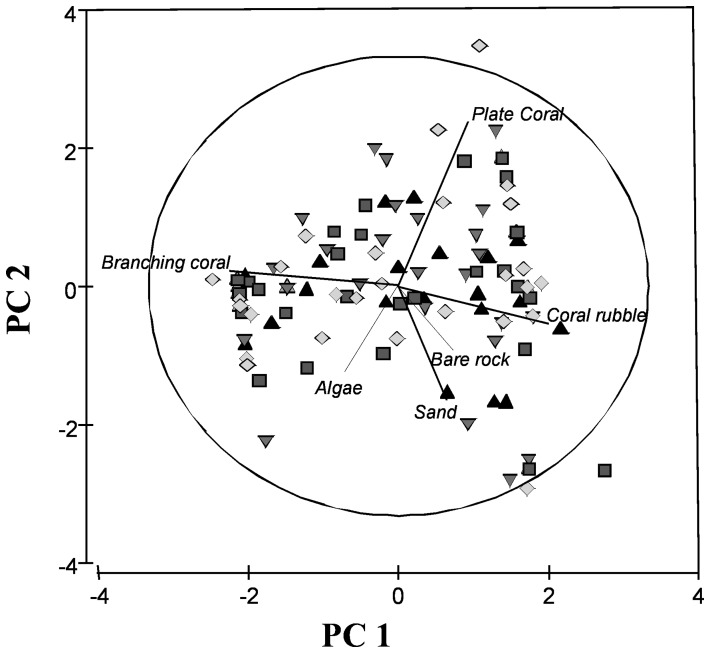
PCA results for habitat. Each point represents a stereo-BRUVs deployment with one of four bait treatments; cat food (triangles), falafel mix (inverted triangles), pilchards (diamonds) and no bait (squares). Habitat contribution to variation is shown with directional vectors.

A long-term monitoring program at the Houtman Abrolhos Islands, Western Australia, investigating the effect of fishing closures has consistently used stereo-BRUVs baited with pilchards (*Sardinops sagax*) [4], [32], [42]–[44]. If the composition or relative abundance of fishes sampled by stereo-BRUVs differs with bait type, this can affect interpretations about the effects of targeted fishery closure management. Therefore, this study examined the use of bait types inside and outside an extensively studied Targeted Fishery Closure (TFC) (Figure 1). The use of pilchards is expected to yield results consistent with previous research while different baits may give different representations of the reef fish assemblage inside and outside the TFC.

The objective of this study was to test whether different bait types (pilchards, cat food, falafel mix and no bait as a procedural control) influenced; (1) the composition of the detected coral reef fish assemblage (assemblage composition, numbers of individuals, number of species and the relative abundance of targeted species), and (2) the consistency of the data produced by the use of different baits inside and outside a TFC. A secondary objective was to compare and contrast the cost and practicality of each type of bait.

## Results

A total of 5209 individuals from 132 fish species belonging to 41 families were recorded by 108 stereo-BRUVs deployments taken from four sites inside and four sites outside the TFC. Samples with no bait (control) recorded a total of 77 species, cat food and falafel mix recorded 76 species while samples with pilchards recorded 75 species. In total, 20 commercially exploited species were identified; 17 by pilchards, 16 by falafel mix, 15 by cat food and 13 by samples with no bait.

### Habitat

A Permutational Multivariate Analysis of Variance (PERMANOVA) on percentage cover of six habitat categories viewed in the video imagery found that habitat was significantly different among bait, fishery status and sites (Table 1). Significant site effects are indicative of high small-scale habitat heterogeneity. Significant status and site effects were investigated using pairwise tests in PERMANOVA. Only within the TFC was habitat between sites significantly different (all P<0.05, except between sites 2 and 4). Compared to sites in the TFC, sites open to fishing had more branching coral and algae and less plate coral.

**Table 1:** PERMANOVA results on habitat percentage cover.

Source	*df*	MS	F	*P*(perm)
Bait	3	62.20	142.49	<0.001
Status	1	123.92	71.68	<0.001
Site (Status)	6	1.76	3.08	<0.001
Bait x Status	3	0.50	1.16	0.32
Bait x Site (Status)	18	0.43	0.75	0.96
Residual	77	0.57		
Total	108			

Results are shown for samples with different bait, status, site (nested within status) and their interactions. Significant values are shown in bold text.

As habitat varied between bait, status and sites, habitat data were used as covariates for the remaining PERMANOVA analyses. PC1 and PC2 explain the effects of different habitat categories that were shown to strongly determine the relationship between habitat and the fish assemblage in a Principle Coordinates Analysis (PCA) (Figure 1). PC1 is correlated to the variability between branching coral and coral rubble vectors, while PC2 is positively correlated to the vector for plate coral and negatively correlated to algae, sand and bare rock vectors (Figure 1). Variation in the relative abundance, the total number of individuals and species richness was significantly associated with habitat variables (all P<0.05). There were no significant interactions between the factors in the experimental design and the habitat covariates, therefore the interaction terms were not included in the models for the remaining PERMANOVA analyses. Using a Distance Based Linear Model (DISTLM), the habitat covariates explained 15.8% of the overall variation in the fish assemblage.

### Assemblage Composition

A Permutational Analysis of Multivariate Dispersions (PERMDISP) showed that the relative abundance was homogenous across bait type and status (P>0.05), but heterogeneous across sites (P<0.01). Data was fourth-root transformed using a Bray-Curtis dissimilarity measure as the raw data was highly variable. The species *Chromis westaustralis* were seen in very high relative abundances within TFCs, and were removed from the dataset to reduce variability and the possibility of high abundance disguising potential relationships and trends in the assemblage data. Using PERMANOVA we detected significant differences in assemblages between different bait types and between different sites (Table 2). Pairwise tests found the difference between bait types was driven by samples with no bait vs. baited treatments (cat food, falafel mix, pilchards) (all P<0.05). The difference in assemblage structure between fishery status was not statistically significant and there were no statistically significant differences detected between interactions with bait, status or site (Table 2).

**Table 2:** PERMANOVA results for relative abundance.

Source	*df*	MS	F	*P*(perm)
PC1	1	11219.00	4.30	**<0.001**
PC2	1	5510.20	2.28	**<0.01**
Bait	3	3076.20	2.51	**<0.001**
Status	1	12253.00	2.08	0.057
Site (Status)	6	5637.90	3.29	**<0.001**
Bait x Status	3	1588.20	1.43	0.08
Bait x Site (Status)	18	1111.70	0.65	1
Residual	75	1712.40		
Total	108			

Relative abundance was recorded in response to samples with different bait, status, site (nested within status) and their interactions. Habitat co-variates (PC1, PC2) were included to observe the significance of the effect of habitat on relative abundance. Significant values are shown in bold text.

Canonical Analysis of Principle Coordinates (CAP) on different bait types supports the PERMANOVA results, where canonical axes separated fish assemblages present at different bait types (P<0.001) and a canonical correlation of δ^2^ = 0.35. The CAP clarifies that there is a significant effect of bait type on the fish assemblage. A PERMDISP on bait type identified variability (mean distance to centroid ± SE) between different samples. Samples with no bait were identified to be the most variable (46.04±1.76), followed by pilchards (43.67±1.31), cat food (42.76±1.14) and falafel mix (42.24±2.16). The leave-one-out allocation table shows the ability of the model to correctly classify the sample to its appropriate group [45]. All groups had relatively low allocation success, with only 39 of 109 samples correctly classified and an overall leave-one-out mis-classification error of 64.22% (Table 3). Cat food and pilchards showed especially low allocation successes, e.g. 41.38% of the cat food was mis-classified as pilchards (Table 3). This indicates that the pilchard and cat food bait sampled similar reef fish assemblages.

**Table 3:** Leave-one-out allocation of observations to groups for overall assemblage composition.

Orig. group	CF	FM	N	P	Total	% correct
Cat food	4	8	5	12	29	13.79
Falafel mix	6	15	2	3	26	57.69
No bait	5	7	15	1	28	53.57
Pilchards	8	7	6	5	26	19.23

**Figure 2:**
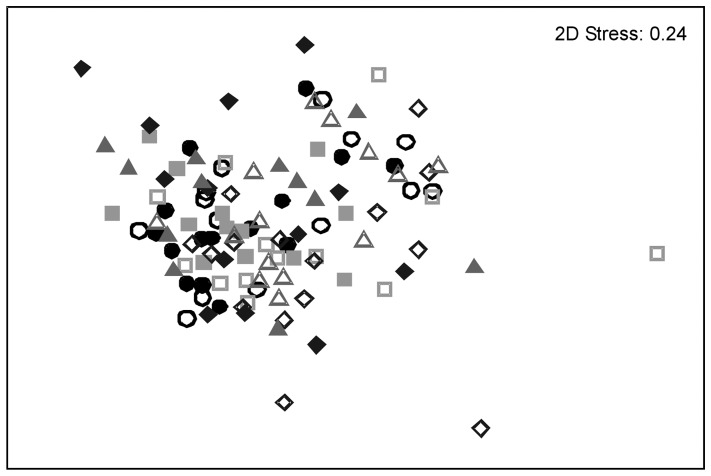
Relative abundance nMDS plot for overall fish assemblage (bait × status interaction). Each point represents an individual sample taken with different bait types; cat food (circles), falafel mix (squares), pilchards (triangles) and no bait (diamonds), which were either inside (closed symbols) or outside (open symbols) the TFC. The species *Chromis westaustralis* was excluded.

**Figure 3:**
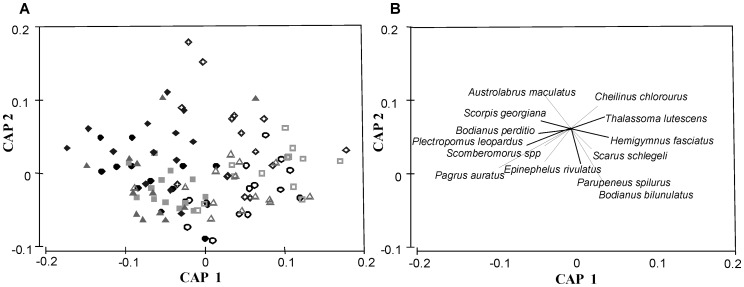
CAP ordination for the complete fish assemblage data set (bait × status interaction). (A) fishery status (y-axis) and bait (x-axis). Each point represents an individual sample taken with different bait types; cat food (circles), falafel mix (squares), pilchards (triangles) and no bait (diamonds), which were either inside (closed symbols) or outside (open symbols) the TFC. The species *Chromis westaustralis* were excluded and the number of axes (m)  = 7. (B) Species contribution to the trends in (A) is shown with directional vectors.

A non-metric Mutidimensional Scaling plot (nMDS) based on Bray-Curtis fourth-root transformed data illustrates the variability of the fish assemblage, where distance between points indicates dissimilarity (Figure 2). Each point on the plot represents a different replicate. The unconstrained ‘bait × status’ nMDS did not show clear effects of bait and status factors, supported by the high 2D stress value (Figure 2). CAP analyses were used to further investigate the bait × status relationship. The CAP plot gives a constrained visual representation of the multivariate data, where canonical axes separated fish assemblages present at different bait types and also distinguished assemblages at TFC vs. fished sites (P  = 0.001) and a large canonical correlation (δ^2^ = 0.61) (Figure 3a). Therefore, the CAP clarifies that there may be a significant effect of the interaction between bait and status by detecting this relationship in higher-dimensional multivariate space. A PERMDISP on bait × status values identified samples with no bait to be the most variable within the TFC (45.19±2.14) and samples with cat food to be the most dispersive in areas open to fishing (42.24±1.22). Samples with falafel were the least variable in both the TFC (37.11±2.24) and areas open to fishing (39.92±2.43). Numerous species contributed to observed differences in fish assemblage structure between bait × status interactions (Figure 3b), indicated by directional vectors corresponding to a Pearson correlation value >0.3.

### Number of Individuals

PERMDISP showed that the number of individuals were homogenous for bait (P>0.05), but heterogeneous for fishery status and site (P<0.05). Data was fourth-root transformed using a Euclidean distance measure. As above, the 8579 *Chromis westaustralis* individuals were removed from the dataset before analysis. PERMANOVA detected significant differences between samples taken with different bait types and among sites (Table 4). The mean number of individuals (average number of individuals ± SE) in samples using pilchards was the highest (51.00±7.13), followed by cat food (42.12±5.14), falafel (41.35±5.71) and no bait (30.68±3.71). Pairwise tests showed the significant difference between bait types was driven by no bait samples in comparison with cat food and pilchard samples. The species complex *Scarus* sp1 (combined species) were the most abundant in all bait types. The next most abundant species were *Pseudocaranx* spp. in both cat food and pilchard samples, and *Plectropomus leopardus* in both falafel mix and no bait samples. There was no statistically significant difference between the number of individuals between fishery status and were no significant interactions between bait, status or site (Table 4).

**Table 4:** PERMANOVA results for the number of individuals.

Source	*df*	MS	F	*P*(perm)
PC1	1	47.03	3.47	**<0.001**
PC2	1	30.30	2.37	**<0.001**
Bait	3	14.14	1.65	**<0.001**
Status	1	49.92	1.83	0.10
Site (Status)	6	26.23	2.67	**<0.001**
Bait x Status	3	9.15	1.13	0.20
Bait x Site (Status)	18	8.09	0.82	0.99
Residual	75	9.81		
Total	108			

5209 individuals were identified in response to samples with different bait, status, site (nested within status) and their interactions. Habitat co-variates (PC1, PC2) were included to observe the significance of the effect of habitat on the number of individuals. Significant values are shown in bold text.

PERMANOVA results for species contributing to the bait-status interaction (*Epinephelus* sp1, *Austrolabrus maculatus*, *Scomberomorus* spp., *Bodianus bilunatus*, *Thalasoma lutescens* and *Scarus* sp1) in the assemblage composition showed a number of significant differences between habitat, bait type, fishery status and site that were species-specific. The relative abundances of *Scarus sp1* and *B. bilunatus* was substantial enough to conduct a General Linear Model (GLM) analysis of variance on length data, however the length of these species did not differ significantly between bait or status factors (P>0.05).

### Species Richness

**Figure 4:**
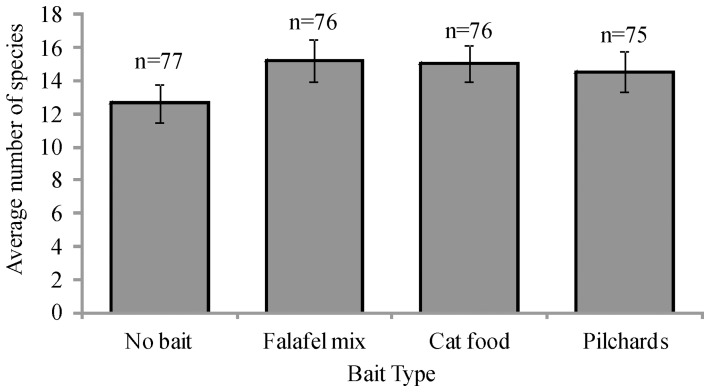
Species richness (y-axis; average number of species ±1 SE) for different types of bait (x-axis). Total number of species (N) is shown for each bait type.

PERMDISP showed that species richness was homogeneous (P>0.05). The results of the PERMANOVA analysis show that there was a strong and significant relationship between habitat and species richness (Table 5). Significant variability was also detected in species richness among sites (Table 5). The average number of species sampled in each stereo-BRUVs deployment varied between bait types, where samples with no bait identified the least number of species on average, but also recorded the most number of species overall (Figure 4). However, the number of species did not differ significantly between different bait types or fishery status and there were no significant interactions between bait, status or site (Table 5).

**Table 5:** PERMANOVA results for the number of species.

Source	*df*	MS	F	*P*(perm)
PC1	1	673.61	11.25	**<0.01**
PC2	1	266.22	5.01	**0.03**
Bait	3	38.023	2.47	0.09
Status	1	69.684	0.40	0.46
Site (Status)	6	169.5	6.17	**<0.001**
Bait x Status	3	8.8545	0.77	0.53
Bait x Site (Status)	18	12.814	0.47	0.96
Residual	75	27.47		
Total	108			

132 fish species were identified in response to samples with different bait, status, site (nested within status) and their interactions. Habitat co-variates (PC1, PC2) were included to observe the significance of the effect of habitat on the number of species. Significant values are shown in bold text.

### Target Species

**Figure 5:**
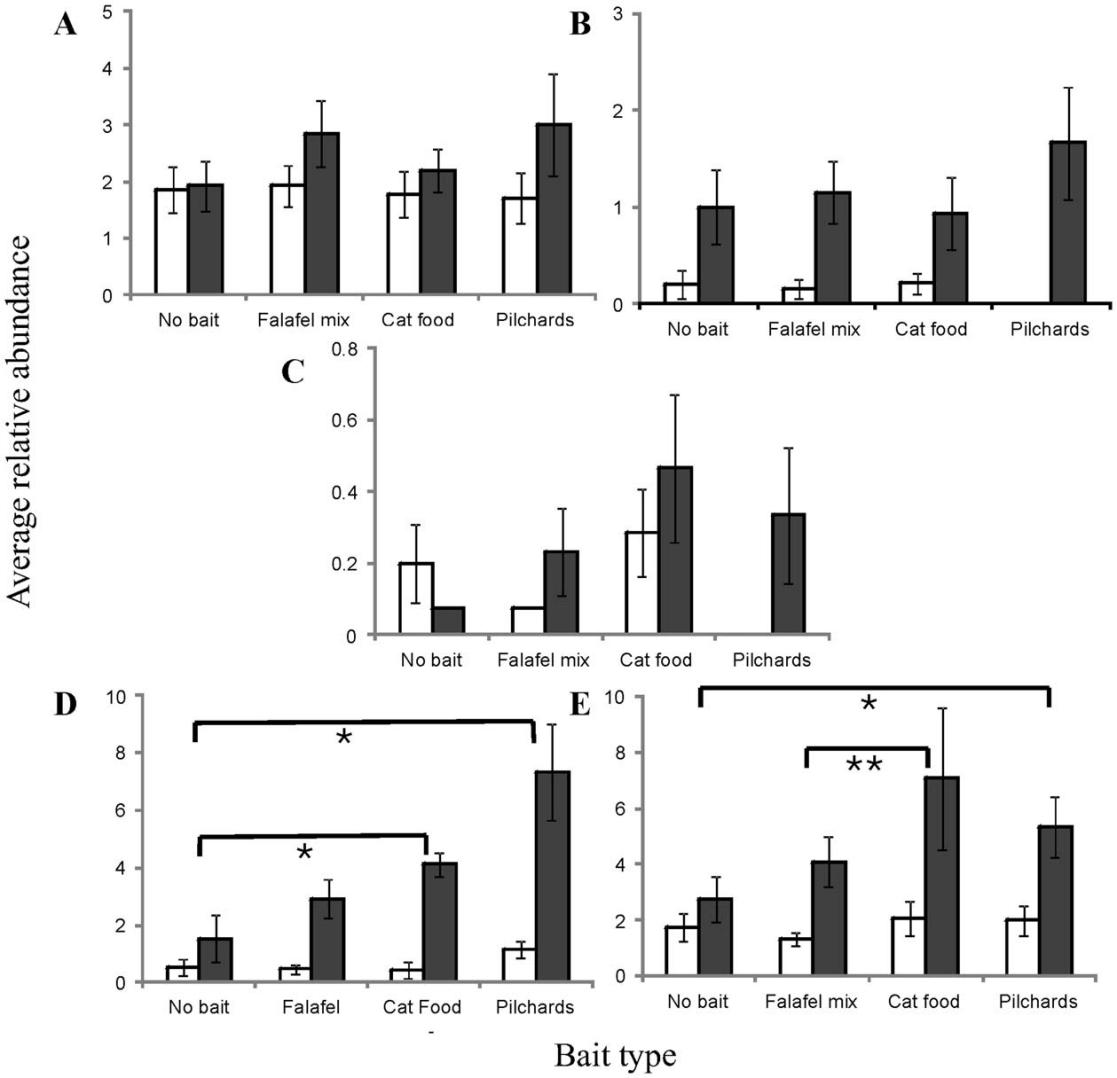
Relative abundance of each of five target species for each of the different types of bait and status. Average relative abundance (y axis; average number of individuals ±1 SE) for different types of bait (x-axis) and status (□  =  Fished, ▪  =  TFC) is shown for (A) *Choerodon rubescens*, (B) *Lethrinus nebulosus*, (C) *Lethrinus miniatus*, (D) *Pagrus auratus* and (E) *Plectropomus leopardus*. Significant differences are shown with (*) where P<0.05 and (**) where P<0.01.

The PERMANOVA results from the abundance of five individual fishery target species showed a number of species-specific significant differences. PERMDISP tests showed all species abundances to be homogeneous across bait, status and sites (all P>0.05) except for *Pagrus auratus* (P<0.01). *P. auratus* was square root transformed while *Choerodon rubescens*, *Lethrinus miniatus*, *Lethrinus nebulosus* and *Plectropomus leopardus* remained untransformed with a Euclidean distance measure. PERMANOVA results for all target species showed significant differences in abundance between habitat categories described by PC1 (all P<0.05). All species except *L. miniatus* differed significantly between sites. *P. leopardus* and *P. auratus* were the only targeted species to differ significantly between bait types (both P<0.05). Pairwise tests revealed the significant difference in abundance of *P. leopardus* between bait types was driven by differences between cat food vs. falafel and pilchards vs. no bait (Figure 5e). The highest abundance of *P. leopardus* was observed from samples obtained with cat food, followed by pilchards, falafel mix and no bait, respectively (Figure 5e).

The only target species to differ significantly in abundance depending on fishery status (P<0.05), and to also exhibit a significant interaction between bait type and fishery status (P<0.05) was *P. auratus*. Pairwise tests showed this interaction was driven by increased relative abundance in samples with cat food and pilchards within the TFC being significantly higher than (1) their corresponding fished sites and (2) TFC samples taken with no bait (Figure 5d). The abundance of all targeted species sampled with all bait types was higher inside the TFC than in sites open to fishing, except for the abundance of *L. miniatus* when sampled with no bait (Figure 5c). GLM analyses on length data of all five targeted species found that length did not differ significantly between bait types (all P>0.05), despite the identification of significantly larger *C. rubescens*, *P. leopardus* and *P. auratus* individuals inside the TFC (all P<0.001).

### Bait Persistence

**Figure 6:**
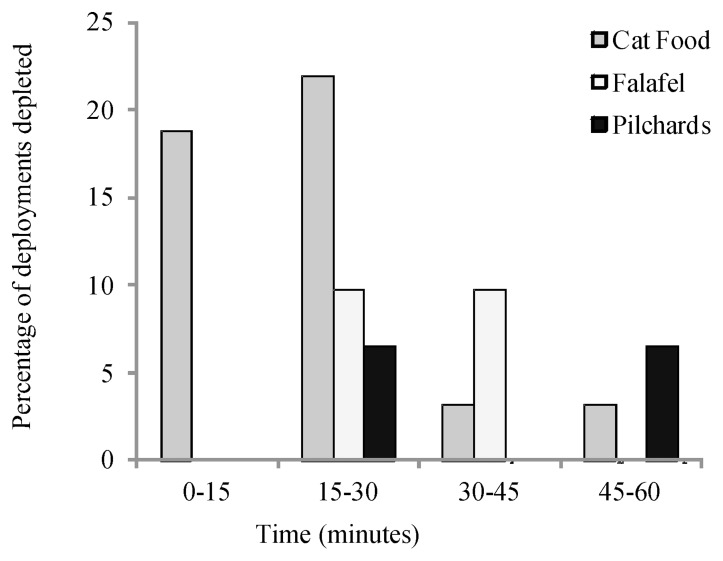
Persistence of different bait types as a percentage of deployments where bait was completely depleted (y-axis), over different time increments during stereo-BRUVs deployment (x-axis). Bait types are represented by grey (cat food), white (falafel mix) and black (pilchards).

Pilchards were the most persistent bait treatment and cat food was the least persistent. Stereo-BRUVs baits that were depleted before full duration of the deployment were evident in 12.9% of deployments with pilchards, 19.4% with falafel mix and 46.9% with cat food. Cat food was the only treatment to completely deplete less than 15 minutes into deployment and in 40.6% of cases was depleted in under 30 minutes (Figure 6). The moisture content of cat food was the highest (82.4%), followed by pilchards (74.0%) and falafel mix (44.1%). This indicates that bait with high moisture content, such as cat food, will not persist over long time periods unless the bait is kept somewhat intact, such as pilchards. Cost analysis demonstrated that stereo-BRUVs deployments baited with falafel mix (AUS $5.87) were more than double the cost of cat food (AUS $2.63) and pilchards (AUS $2.41), which were similar in price to each other.

## Discussion

### Assemblage Composition

The significant differences in the assemblage composition between the types of bait was driven by increases in the relative abundance of fish in baited compared to non-baited stereo-BRUVs, regardless of the type of bait. This was expected, as it is consistent with comparative stereo-BRUVs studies [2], [4], [14], [15] and trapping literature globally [5], [19], [28], [46], [47]. The difference between baited and non-baited samples is attributed to an increased presence of species that are attracted to the bait, which are typically species employing predatory or scavenging feeding strategies [2]. A conspecific attraction effect was also observed during video analysis, where individuals who first approached the bait would also attract other conspecifics. This behaviour has been observed previously with stereo-BRUVs [15]. Fish were also observed approaching the stereo-BRUVs for reasons other than the pursuit of bait, such as random movements, curiosity, intraspecific social behavior (conspecific attraction) and predatory behavior, which have been observed as factors contributing to the capture success of fish traps [30]. Unlike non-baited stereo-BRUVs, the video ‘capture’ of any species attracted to bait is no longer left primarily to chance [5], thereby increasing both the relative abundance in baited stereo-BRUVs and the higher variability of fish assemblages recorded with non-baited stereo-BRUVs. It is also noteworthy that the ‘leave-one-out’ analysis incorrectly classified a large portion of cat food samples as pilchard samples, indicating that they may be similar in their ability to sample reef fish assemblages (Table 3). This is likely to be attributable to the cat food containing pilchard and trevally cutlets (the exact species were not specified), which would have similar attracting properties to pilchards alone. In terms of assemblage composition, these results indicate that cat food may suffice as an alternative to pilchards in areas where pilchards may not be available for use as bait.

### Number of Individuals

Like the assemblage composition, results for the overall number of individuals were consistent with previous studies where non-baited samples had significantly less individuals recorded than baited samples [2], [15], [19], [28], [46]. In this study, this difference was driven by cat food and pilchard samples in comparison to non-baited samples only. Samples with falafel mix were not significantly different to any other bait treatment, which is contrary to a pilot study that has determined falafel mix to attract noticeably higher abundances than pilchards in east Australian waters. Pilchards recorded an average of 51 individuals per stereo-BRUVs deployment in this study (temperate-tropical), while Wraith [41] reported an average of 45 individuals in New South Wales, Australia (temperate) and Cappo et al. [14] reported an average of 29 individuals in northern Australia (tropical). This highlights the need to carry out preliminary experiments to determine which type of bait will be most practical for use in the area of study, as the use of bait with stereo-BRUVs can discriminate fish assemblages in tropical and temperate environments [2]. Different bait types may also be applicable directly to the aims of different experiments focusing on different trophic groups.

Using bait to measure the number of individuals is advantageous as more individuals of a single species are attracted closer to the camera, providing better opportunities for identification and accurate length measurements [2]. It is acknowledged that variation in the attraction of fish to the bait is often species-specific [2], [19]. The abundance of individual fishes and the number of species at baited gear depends upon a range of stimuli such as chemosensory abilities, feeding motivation, search pattern, activity, schooling behaviour and the response time of the individual [2], [48]–[50]. An example of species-specific effects on sampling is the visual stimuli of the bait, which has been documented to strongly influence catch rates in long-lining gears [23]. Harvey et al. [2] state that stereo-BRUVs are likely to introduce biases not associated with extractive fishing gears because of the multiplicity of behaviours adopted by reef fishes. Hence, caution is required when drawing conclusions from the variation in fish assemblages sampled with different baits because of the unidentified behavioural response of many species.

Differences in the sampling efficiency of different bait types are due to their physical properties, such as consistency and therefore bait plume capabilities [19], [26]. The high numbers of individuals attracted by cat food and pilchards may suggest that these bait types have good bait plume capabilities (e.g. high moisture content and thus dispersive capacity). Further factors to consider include the maceration of the bait by the first arriving species, the “chumming effect” (increasing odour leaching and thus attracting more species) and the influence of benthic topography and current velocities on the speed and direction of the bait plume [49], [51]. It would be advantageous to measure these qualities for the different bait treatments in this study, however modeling bait plumes for benthic baited techniques is very complex and relies on many assumptions [33], [52]. Additionally, it has been confirmed that most fish species tend to approach a baited structure from down-current [8], [19], therefore both behavioural effects and the directional influence of oceanography on the plume is also of significance. Further research is required to identify what processes may be driving some species towards particular bait types.

### Species Richness

Species richness data recorded by the different types of bait during this study did not follow the pattern found by other comparative BRUV studies, which report that the use of bait increased species diversity as well as the relative abundance of fishes [2], [4], [14], [15], [28]. Harvey et al. [2] found that the number of species increased significantly in baited stereo-BRUVs, but the number of species from herbivorous trophic groups were the same as non-baited stereo-BRUVs. Observation of fish behaviour during this video analysis suggested that small and cryptic fishes were attracted to the falafel mix and cat food, as they could feed on particles rising from the bait bag. The damselfish, *Chromis notata*, has also been observed demonstrating this behaviour surrounding an underwater video baited with fish mince in southern Japan [28]. The attraction of small, cryptic fishes to this type of bait may be the reason for the lack of significant differences in species richness between bait types including the baited vs. non-baited effect. Results from this study suggest that non-baited stereo-BRUVs would be just as efficient as baited stereo-BRUVs in measuring fish species richness at the Houtman Abrolhos Islands.

### Site Variation

Between-site differences were the most important component of variability in the assemblage composition, number of individuals and species richness, indicating high small-scale variability (Table 2, 4, 5). A considerable proportion of site variation was caused by habitat heterogeneity, which has consistently been found to affect fish assemblage structure [35], [53]–[57]. Local habitat characteristics and the patchiness of local structures generate habitat heterogeneity in marine ecosystems, for example macrophyte occurrence can influence species occurrence at small spatial scales [48], [58]. Fish behaviour may also contribute to variability between sites, such as mobility and feeding strategy [15]. Habitat information from towed video camera [14], [59] and towed diver surveys [60], [61] can be utilized in planning a sampling design to minimize habitat heterogeniety. In this study the inclusion of habitat as covariates accounted for the site-by-site variation, the consistent insignificance of bait-site interactions indicates that site variability does not mask or confound any effects between bait types or fishery status.

### Targeted Fishery Closure Effects

There were no significant differences in the number of individuals or species richness between the TFC and areas open to fishing between different bait types. This was expected for species richness as it may not be a sensitive indicator of closure effectiveness as a result of uncommon species that are only occasionally observed [62]. However, the overall number of individuals was expected to be significantly higher within the TFC, consistent with previous research [15], [32]. Possible reasons for the lack of a closure effect are not known, but assumption that the behavioural response of fish to bait is the same inside and outside the TFC.

### Target Species

Target species are of particular interest at the Houtman Abrolhos Islands because of their vulnerability to commercial and recreational fishing [63] and the uncertain response of these species to protection from fishing [43], [44]. Relative abundances of all five chosen target species (*Pagrus auratus*, *Choerodon rubescens*, *Lethrinus miniatus*, *Lethrinus nebulosus* and *Plectropomus leopardus*) differed significantly according to branching coral and coral rubble habitat, but were not affected by other habitat categories. The relative abundances of all target species except *L. miniatus* differed significantly between sites. Significant variability in abundance between sites was expected for highly mobile species, including *L. miniatus*, similar to that reported by McLean et al. [43].

Any descriptions of *P. leopardus* and *P. auratus* abundance made with stereo-BRUVs are potentially confounded by the effect of bait type (Figure 5), with differences between locations possibly due to the type of bait and its ability to sample representatively. *P. leopardus* were never observed to feed directly on the bait, however some individuals were observed feeding on particles rising from the bait and were considered ‘attracted’. In addition, the relative abundance of *P. auratus* supported the hypothesis that there may be differences in the data set produced by sampling with different bait types inside and outside the TFC. The absence of other target species response to protection could result from a range of factors (see [43], [44], [64]). The lengths of *P. auratus*, *C. rubescens* and *P. leopardus* indicate that the size of targeted species will not differ significantly according to the type of bait used. However, the size of these species do respond to protection from fishing, which supports previous research from the Houtman Abrolhos Islands [32], [42] and elsewhere [7].

### Bait Practicality

Bait practicality was assessed through investigation of persistence and expense. Pilchards were the most persistent, providing further justification for extensive use with commercial fish trap fisheries that often deploy bait for many consecutive hours [18], [30]. However, cat food was depleted earlier and more often than pilchards and the falafel mix. Schools of *P. auratus* were observed to be responsible for the rapid depletion of cat food the majority of the time. Cat food for use as bait may be more practical when used in marine environments where schooling species such as *P. auratus* are not present, such as around Guam, where fish diversity and abundance are relatively low [65]. The lack of persistence in cat food is also probably attributed greatly to its extremely high moisture content in combination with its fleshy consistency. Catch rates in fish traps are higher when there is consumption and thus loss of bait [3], [51]. This may explain why cat food continued to attract a greater abundance of fishes than falafel mix, which had less than half the moisture content of cat food.

Previous studies suggest that approximately 25–30 minutes is sufficient duration for BRUVs and stereo-BRUVs deployment [8], [14]. It is also known that fish arrivals to baited video methods decrease with a loss of bait [19], therefore bait loss before completion of deployment does compromise stereo-BRUVs samples. Given that cat food was found by CAP analyses and leave-one-out allocation to be similar to pilchards (Figure 3a, Table 3), it may have potential as stereo-BRUVs bait despite its inability to consistently sample the full hour. This result suggests that cat food may have the ability to sample significantly higher abundances than pilchards if changes are made to the delivery of the bait so that improved bait persistence is accomplished. Improvements to cat food initially would be to increase the amount of bait used, but this would increase cost. Reducing accessibility of fishes to the bait would also increase persistence, which could be achieved with bait bags of a smaller mesh size or the construction of ‘teabag’ type bags using stockings in a protective mesh covering, as seen with fish mince in Archdale et al. [28].

In terms of cost, cat food is similar to pilchards (approx. AUS $2.50 per stereo-BRUVs deployment) and less than half the price of falafel (approaching AUS $6.00 per stereo-BRUVs deployment). However, the cost of bait for stereo-BRUVs is marginal in comparison to other logistical costs and to other sampling methods, for example, Brooks et al. [66] found that long-lining surveys consumed 92.1% more bait than baited video methodology over the course of the study. However, bait sustainability and the subsequent environmental cost of the bait needs to be taken into consideration, where the use of tuna oil in falafel mix, although only a small portion, substantially decreases its environmental and economic sustainability [67]. In addition, the use of untreated, frozen fish products that have been transported across geographic boundaries carries a risk of spreading disease, for example the mass mortalities of pilchards in 1995 and 1998/99 in Australia (e.g. [68] or the introduction of white-spot syndrome virus through imported shrimp in the Gulf of Mexico [69]. Cat food has been heat treated or irradiated when it arrives in Australia [70] so any risk of disease when deployed in the marine ecosystem would be extremely low.

Furthermore, falafel mix created a bait plume when disturbed that greatly reduced visibility and hindered species identification. Cat food also produced a visible plume, although this did not hinder species identification or measurements as frequently as falafel mix. Falafel mix also was not practical during transportation, as it had to remain frozen solid on long trips to avoid odours and mess whereas cat food was the most easily transported. Further research is needed to investigate cat food as a prospective stereo-BRUVs bait, as it could contribute greatly to monitoring programs at the Houtman Abrolhos Islands and elsewhere. However, the use of pilchards as stereo-BRUV bait is practical and does not require any improvements.

### Conclusions

The results of the present study provide evidence in support of the hypothesis that different bait types may have an effect on sampling the structure of reef fish assemblages, in terms of abundance and assemblage composition. The relative abundance of reef fishes did differ significantly in areas open versus closed to fishing when different bait types were used. The abundance of the highly targeted *P. auratus* indicated that there is an advantage of using pilchards or cat food as bait in monitoring programs for this species. Given that pilchard baits result in consistent numbers of fish among samples (less variation), sample similar assemblages, exhibit higher means among sites and were persistent (long lasting) indicate that the use of pilchards in conjunction with stereo-BRUVs is justified on the mid-west coast of Western Australia and potentially elsewhere. However, the results of this study also indicate that cat food (as an alternative to pilchards) could also be an acceptable bait treatment once complications with persistence are addressed. In order to sample a comprehensive and adequate proportion of reef fish assemblages, preliminary studies should be undertaken between different types of bait. Overall, this study has revealed that different types of bait are capable of sampling different components of reef fish assemblages, thus highlighting the need for the standardisation of bait types over time and between studies so that effective comparisons can be undertaken.

## Methods

### Site Description

**Figure 7:**
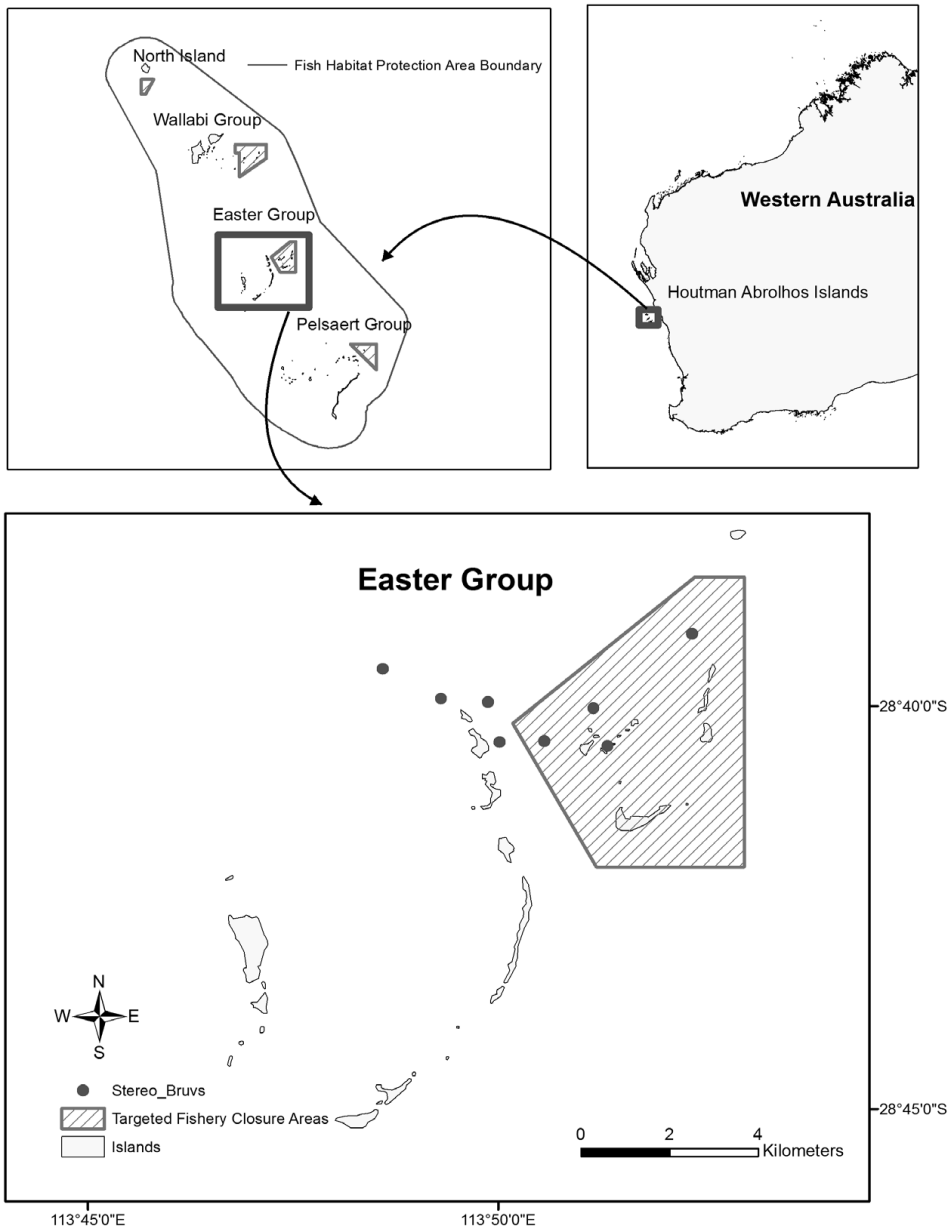
Houtman Abrolhos Islands, Western Australia. The location of each island group and closed areas (Targeted Fishery Closure Areas) are shown, including 8 site locations (Stereo-BRUVs) in which 4 replicates of each bait type were deployed.

Sampling was conducted with stereo-BRUVs inside and outside of a Targeted Fishery Closure (TFC) at the Houtman Abrolhos Islands, Western Australia. The Houtman Abrolhos Islands are approximately 60 kms offshore of the mid-west coast of Western Australia (Figure 7). The reef habitats of the Houtman Abrolhos Islands differ from most other coral communities in that they are mixed with fleshy macro-algal species, which is attributed to the temperate-tropical transitional composition of the area [32], [71]. As such, there is a great diversity of reef fish, including both temperate and tropical species [71]. TFCs were established in 1994 to protect reef fish that are vulnerable to exploitation (Figure 7). This study was conducted within the TFC and corresponding control locations at the Easter group of the Abrolhos Islands.

### Experimental Design

The sampling design consisted of three factors: Bait (four levels; pilchards, cat food, falafel mix and no bait (control); fixed), Status (two levels; TFC and fished area; fixed), and Site (four levels, nested in Bait x Status; random). Reef fish assemblages were observed at each site over four consecutive days using baited remote underwater stereo-video systems (stereo-BRUVs). Each stereo-BRUVs deployment location was standardised for depth (15–30 m) and substrate (coral reef), using direct observation, echo-sounder readings and the skippers’ knowledge. Samples were collected during daylight hours, allowing an hour between sampling and sunrise/sunset to avoid possible crepuscular variation in fish assemblages. For each bait type, four replicate stereo-BRUVs were deployed at each of the four sites (inside and outside the TFC). The type and location of bait within each site were determined randomly, so that each bait was replicated a total of 32 times (4 in each site, 16 in each status), resulting in a total of 128 samples. However, samples where the stereo-BRUVs tilted upwards and habitat could not be analysed were removed from the dataset, resulting in only 108 samples being available for statistical analysis. Replicate stereo-BRUVs deployments were placed randomly within each site, with no replicate being deployed consecutively at the same site. A minimum distance of 250 m was kept between stereo-BRUVs deployments to avoid overlap of bait plumes and to reduce the likelihood of fish moving between sites during the sampling period [14], [32].

Comparisons were made between a control treatment (no bait) and three different bait types differing in consistency; pilchards (*Sardinops sagax*), cat food (pilchard & trevally cutlets) and falafel mix combined with tuna oil and water (herein referred to as ‘falafel mix’). Pilchards were purchased frozen and remained frozen until the day before use. They were then crushed to maximise the dispersal of fish attracting oils in mesh bags directly before use. Cat food was transported in cans, and emptied into mesh bait bags directly before use. Falafel mix was prepared by combining 5 kg of falafel mix with 1 L concentrated tuna oil and 4 L water, frozen into containers and then transferred to mesh bait bags to defrost during stereo-BRUVs deployment. For uniformity, each treatment consisted of approximately 800 g of bait, a quantity that is based on previous studies that have found stereo-BRUVs to be a comprehensive sampling method [4], [15], [72]. Bait was replaced after every sample.

### Sampling Equipment

Two Sony CX12 full high definition cameras were supported in camera housings 0.7 m apart on the base bar of a trapezium-shaped galvanised steel frame. The cameras were inwardly converged at 8 degrees to gain a maximum field of view and to allow for three dimensional calibration used for fish length measurements [32]. A plastic mesh basket containing the various bait treatments was suspended 1.2 m in front of the cameras [15]. Once deployed and settled on the seafloor, the stereo-BRUVs recorded 60 minutes of footage, based on previous research outlining minimum deployment times [5], [73].

### Video Analysis

#### Calibration

Xilisoft video conversion software was used to convert from MT2S to AVI format to facilitate image analysis. The program CAL (SeaGIS Pty. Ltd.) was used to calibrate stereo-BRUVs before and after completion of the field work in order to make accurate measurements of the sampling area and fish length [74]. This process is described in detail by Harvey & Shortis [75].

#### Image analysis

The software ‘EventMeasure (Stereo)’ (SeaGIS Pty. Ltd.) was used to identify and quantify species and abundance, while also measuring the length of individuals [76]. To avoid repeat counts of individual fish continuously re-entering the field of view, the maximum number of individuals of the same species appearing at the same time (MaxN) was used as a relative abundance measure. MaxN is a conservative estimate of relative abundance, which is essential as the variability in attraction of the bait has the potential to overestimate abundance [2], [13], [14], [77]. If the bait was depleted before the end of the sampling timeframe, the time of depletion was noted during video analysis. The percentage of samples from each type of bait that were depleted before the full stereo-BRUVs deployment was used as a measure of bait persistence. Length measurements of fishes is fork length in millimeters and can be used to determine the relative size structure of fish assemblages [76].

In this research, 91.3% of species sighted could be identified to the species level. One limitation of video analysis with stereo-BRUVs was that occasionally it was not possible to identify species due to impaired lighting or a restricted field of view of the cameras. Where visual differences between species could not be identified, the MaxNs were combined in the raw dataset. Species that could not be separated included *Epinephelus fasciatus* with *Epinephelus rivulatus* (termed *Epinephelus* sp1 in the analyses) and *Chlorurus sordidus* with *Scarus schlegeli* (termed *Scarus* sp1 in the analyses).

#### Habitat

The software Dots on Rocks (DOR) was used to analyse habitat images obtained from the beginning of every stereo-BRUVs deployment in EventMeasure (Stereo). Six habitat categories were assessed and the percentage cover was calculated. These habitat categories were: plate coral species, branching coral species, coral rubble, exposed rock, sand and macroalgae/kelp. DOR was used to assign 30 randomised points on 5×3 grid over each habitat image and each point was then allocated into a habitat category.

### Moisture Content and Cost

The moisture content of the each different bait type was determined by weighing an 800 g sample of each before and after oven-drying in an industrial oven for 144 hours at 100°C. Loss of mass over time indicates moisture content and was calculated as a percentage of the total bait sample.

The total cost was calculated for each bait type and divided by the total number of drops (32) to determine the cost per stereo-BRUVs deployment (AUD). Logistical costs of the stereo-BRUVs deployment itself were not included as they were identical between bait treatments.

### Data Analysis

Permutational multivariate analysis of variance (PERMANOVA) [78] was used to analyse habitat percent cover and relative abundance data sets using the PERMANOVA + add on to Plymouth Routines In Multivariate Ecological Research (PRIMER-e) package [45], [79].

#### Habitat

Habitat percentage cover was arcsine transformed into degrees to normalize possible binomial distributions, a common characteristic of proportional data [80]. Euclidean distance dissimilarity matrix was used for the subsequent PERMANOVA analysis [81]. Habitat variables were also included as covariates in the PERMANOVA analyses of the assemblage, abundance and species richness data sets. This was achieved with a PCA on the habitat percentage cover (in degrees) where habitat points were projected perpendicularly onto axes that minimise residual variation in Euclidean space [45]. Values taken by individual samples along each of the two PCA axes (PC1 and PC2 scores) were exported from the analyses and used as habitat covariates. Vectors explaining the effect of each habitat category on the construction of the constrained ordination picture were strong in directionality along PC1 and PC2, therefore both axes were used as covariates. The analyses using habitat as a covariate required the use of Type I sequential sums of squares [79]. Percentage variation of the assemblage, abundance and species richness explained by habitat was calculated using a distance-based analysis on a linear model DistLm with 9999 permutations within the PRIMER-e.

#### Assemblage Composition

The relative abundance, as measured by MaxN, describes the overall fish assemblage composition. Fish assemblage data was analysed using a three-factor (bait, fishery status and site) non-parametric PERMANOVA. A Bray-Curtis coefficient on fourth root transformed data was chosen because the data was highly variable (0 to >250) and its value is unchanged by joint species absence from samples, among other desirable criteria [79]. The fourth root transformation allowed both mid-range and rarer species to exert some influence on the calculation of similarity [79]. Significance values were obtained by computing 9999 permutations of the raw data units for each term in the analysis. Pairwise comparisons were then used between variables to determine where significant differences occurred within the fish assemblage [82]. Permutational Analysis of Multivariate Dispersions (PERMDISP) was used to test for differences in multivariate dispersions, for groups of bait, fishery status and site. Non-metric Multidimensional Scaling (nMDS) plots were produced to illustrate the variability present in the fish assemblage, through visualising the dissimilarities between factors.

Canonical Analysis of Principle Coordinates (CAP) was used to test for differences between groups of significant factors, to illustrate patterns often masked by unconstrained nMDS plots and to identify those species primarily responsible for dispersions [83], [84]. Spearman rank correlations were used to produce vector overlays on CAP plots, to highlight the overall increasing or decreasing relationships of individuals variables across the plot [45]. Bait and bait-status relationships were further investigated with CAP analyses. Eigen values (δ^2^) of the CAP ordinations are the square of the canonical correlations and provide an indication of the strength of the observed differences among treatments in the data set in relation to an axis. CAP analyses also generated leave-one-out allocation success. The number of axes (m) was chosen by plotting the residual sum of squares. The first significant drop in relation to the other values was chosen which results in a minimum mis-classification error [83]. Species contributions to trends detected in the CAP are shown with directional vectors.

#### Species Richness, Numbers of Individuals & Length

Univariate PERMANOVAs were used to detect trends in abundance and species richness data using the process described above except that a Euclidean distance measure on fourth root transformed (abundance) and untransformed (species richness, targeted species, length) data was used. Numbers of individuals (abundance) refers to the total number of individuals, regardless of species, identified cumulatively across bait types, status or site. Five commercially exploited species, hereon termed ‘target’ species, were chosen for analysis because they are of recreational and commercial importance at the Houtman Abrolhos Islands. These target species are; *Choerodon rubescens, Lethrinus miniatus, Lethrinus nebulosus, Pagrus auratus* and *Plectropomus leopardus*, and have been documented to occur in significantly higher abundance within the TFC [32]. Data was tested for homogeneity using PERMDISP, which is equivalent to Levene’s test for heterogeneity of variances when used on univariate data [82]. Fish length data was investigated using a General Linear Model (GLM) for targeted species and species of interest in the program Minitab V 13. The factors ‘bait’, ‘status’ and the ‘bait × status’ interaction were used in the GLM. Where length measurements could not be obtained for every ‘bait × status’ combination, length data was analysed for ‘bait’ and ‘status’ factors only.

## References

[1] Holbrook SJ, Kingsford MJ, Schmitt RJ, Stephens JS (1994) Spatial and temporal patterns in assemblages of temperate reef fish. American Zoologist 34: 463–475.

[2] Harvey ES, Cappo M, Butler JJ, Hall N, Kendrick GA (2007) Bait attraction affects the performance of remote underwater video stations in assessment of demersal fish community structure. Marine Ecology Progress Series 350: 245–254.

[3] Cappo M, Harvey ES, Shortis M (2006) Counting and measuring fish with baited video techniques - an overview. Australian Society for Fish Biology: Workshop Proceedings. 101–114.

[4] Watson DL, Harvey ES, Fitzpatrick BM, Langlois TJ, Shedrawl G (2010) Assessing reef fish assemblage structure: how do different stereo-video techniques compare? Marine Biology 157: 1237–1250.

[5] Stobart B, Garcia-Charton J-A, Espejo C, Rochel E, Goni R, et al (2007) A baited underwater video technique to assess shallow-water Mediterranean fish assemblages: Methodological evaluation. Journal of Experimental Marine Biology and Ecology 345: 158–174.

[6] Ellis DM, DeMartini EE (1995) Evaluation of a video camera technique for indexing abundances of juvenile pink snapper, *Pristipomoides filamentosus*, and other Hawaiian insular shelf fishes. Fishery Bulletin 93: 67–77.

[7] Babcock RC, Kelly S, Shears NT, Walker JW, Willis TJ (1999) Changes in community structure in temperate marine reserves. Marine Ecology Progress Series 189: 125–134.

[8] Willis TJ, Babcock RC (2000) A baited underwater video system for the determination of relative density of carnivorous reef fish. Marine and Freshwater Research 51: 755–763.

[9] Willis TJ, Millar RB, Babcock RC (2000) Detection of spatial variability in relative density of fishes: comparison of visual census, angling, and baited underwater video. Marine Ecology-Progress Series 198: 249–260.

[10] Langlois T, Chabanet P, Pelletier D, Harvey ES (2006) Baited underwater video for assessing reef fish populations in marine reserves. SPC Fisheries Newsletter. 53–57.

[11] Cappo M, De' ath G, Speare P (2007) Inter-reef vertebrate communities of the Great Barrier Reef Marine Park determined by baited remote underwater video stations. Marine Ecology Progress Series 350: 209–221.

[12] Priede IG, Merrett NR (1996) Estimation of abundance of abyssal demersal fishes; a comparison of data from trawls and baited cameras. Journal of Fish Biology 49: 207–216.

[13] Stewart BD, Beukers JS (2000) Baited technique improves censuses of cryptic fish in complex habitats. Marine Ecology Progress Series 197: 259–272.

[14] Cappo M, Speare P, De' ath G (2004) Comparison of baited remote underwater video stations (BRUVS) and prawn (shrimp) trawls for assessments of fish biodiversity in inter-reefal areas of the Great Barrier Reef Marine Park. Journal of Experimental Marine Biology and Ecology 302: 123–152.

[15] Watson DL, Harvey ES, Anderson MJ, Kendrick GA (2005) A comparison of temperate reef fish assemblages recorded by three underwater stereo-video techniques. Marine Biology 148: 415–425.

[16] Stoner AW, Laurel BJ, Hurst TP (2008) Using a baited camera to assess relative abundance of juvenile Pacific cod: Field and laboratory trials. Journal of Experimental Marine Biology and Ecology 354: 202–211.

[17] MacRae PSD, Jackson DA (2006) Characterizing north temperate lake littoral fish assemblages: a comparison between distance sampling and minnow traps. Canadian Journal of Fisheries and Aquatic Sciences 63: 558–568.

[18] Anon (1987) Development of more efficient traps for the north-west shelf fishery. Western Australia: Department of Fisheries.

[19] Whitelaw AW, Sainsbury KJ, Dews GJ, Campbell RA (1991) Catching characteristics of four fish-trap types on the North West Shelf of Australia. Australian Journal of Marine and Freshwater Research 42: 369–382.

[20] Saila SB, Nixon SW, Oviatt CA (2002) Does lobster trap bait influence the Maine inshore trap fishery? North American Journal of Fisheries Management 22: 602–605.

[21] Smith PA (2002) The relationship between stock and catch and the effect of bait on catch as determined for a UK recreational catch and release fishery. Fisheries Management and Ecology 9: 261–266.

[22] Grixti D, Conron SD, Jones PL (2007) The effect of hook/bait size and angling technique on the hooking location and the catch of recreationally caught black bream *Acanthopagrus butcheri* . Fisheries Research 84: 338–344.

[23] Broadhurst MK, Hazin FHV (2001) Influences of type and orientation of bait on catches of swordfish (*Xiphias gladius*) and other species in an artisanal sub-surface longline fishery off northeastern Brazil. Fisheries Research 53: 169–179.

[24] Lowry M, Steffe A, Williams D (2006) Relationships between bait collection, bait type and catch: A comparison of the NSW trailer-boat and gamefish-tournament fisheries. Fisheries Research 78: 266–275.

[25] Sainte-Marie B, Hargrave BT (1987) Estimation of scavenger abundance and distance of attraction to bait. Marine Biology 94: 431–443.

[26] Sheaves MJ (1995) Effect of design modifications and soak time variations on antillean-z fish trap performance in a tropical estuary. Bulletin of Marine Science 56: 475–489.

[27] Cowx IG, Gerdeaux D (2004) The effect of fisheries management practises on freshwater ecosystems. Fisheries Management and Ecology 11: 145–151.

[28] Archdale MV, Anasco CP, Tahara Y (2008) Catches of swimming crabs using fish mince in "teabags" compared to conventional fish baits in collapsible pots. Fisheries Research 91: 291–298.

[29] Jones JB, Gibson AP (1997) Risk analysis for the practice of importing frozen fish as bait. Western Australian Fishing Industry Council (Inc.). Perth, Western Australia.

[30] Newman SJ, Skepper CL, Mitsopoulos EA, Wakefield CB, Meeuwig JJ, et al (2011) Assessment of the potential impacts of trap usage and ghost fishing on the northern demersal scalefish fishery. Reviews in Fisheries Science 19: 74–84.

[31] Denny CM, Babcock RC (2003) Do partial marine reserves protect reef fish assemblages? Biological Conservation 116: 119–129.

[32] Watson DL, Harvey ES, Kendrick GA, Nardi K, Anderson MJ (2007) Protection from fishing alters the species composition of fish assemblages in a temperate-tropical transition zone. Marine Biology 152: 1197–1206.

[33] Heagney EC, Lynch TP, Babcock RC, Suthers IM (2007) Pelagic fish assemblages assessed using mid-water baited video: standardising fish counts using bait plume size. Marine Ecology Progress Series 350: 255–266.

[34] Malcolm HA, Gladstone W, Lindfield S, Wraith J, Lynch TP (2007) Spatial and temporal variation in reef fish assemblages of marine parks in New South Wales, Australia - baited video observations. Marine Ecology-Progress Series 350: 277–290.

[35] Moore CH, Harvey ES, Van Niel KP (2009) Spatial prediction of demersal fish distributions: enhancing our understanding of 4 species-environment relationships. ICES Marine 66: 2068–2075.

[36] Moore CH, Harvey ES, Van Niel KP (2010) The application of predicted habitat models to investigate the spatial ecology of demersal fish assemblages Marine Biology. 157: 2717–2729.

[37] Moore CH, Van Niel KP, Harvey ES (2011) The effect of landscape composition and configuration on the spatial distribution of temperate demersal fish. Ecography 34: 425–435.

[38] Chatfield BS, Van Niel KP, Kendrick GA, Harvey ES (2010) Combining environmental gradients to explain and predict the structure of demersal fish distributions. Journal of Biogeography 37: 593–605.

[39] Monk J, Ierodiaconou D, Bellgrove A, Harvey E, Laurenson L (2011) Remotely sensed hydroacoustics and observation data for predicting fish habitat suitability. Continental Shelf Research 31: S17–S27.

[40] Murphy HM, Jenkins GP (2010) Observational methods used in marine spatial monitoring of fishes and associated habitats: a review. Marine & Freshwater Research 61: 236–252.

[41] Wraith JA (2007) Assessing reef fish assembalges in a temperate marine park using baited remote underwater video. 56 pg.: University of Wollongong. 56 p.

[42] Watson DL, Anderson MJ, Kendrick GA, Nardi K, Harvey ES (2009) Effects of protection from fishing on the lengths of targeted and non-targeted fish species at the Houtman Abrolhos Islands, Western Australia. Marine Ecology-Progress Series 384: 241–249.

[43] McLean DL, Harvey ES, Fairclough DV, Newman SJ (2010) Large decline in the abundance of a targeted tropical lethrinid in areas open and closed to fishing. Marine Ecology-Progress Series 418: 189–199.

[44] McLean DL, Harvey ES, Meeuwig JJ (2011) Declines in the abundance of coral trout (*Plectropomus leopardus*) in areas closed to fishing at the Houtman Abrolhos Islands, Western Australia. Journal of Experimental Marine Biology and Ecology 406: 71–78.

[45] Anderson MJ, Gorley RN, Clarke KR (2008) PERMANOVA+ for PRIMER: Guide to Software and Statistical Methods. PRIMER-E, Plymouth, UK.

[46] Munro JL (1974) The mode of operation of Antillean fish traps and the relationships between ingress, escapement, catch and soak. ICES Journal of Marine Science 35: 337–350.

[47] Newman SJ (1990) Effects of depth of set, mesh size, bait and lunar phase on the performance of fish traps on the Great Barrier Reef. Townsville: BSc (Hons) thesis. James Cook University of North Queensland, Townsville.

[48] Alos J, Arlinghaus R, Palmer M, March D, Alvarez I (2009) The influence of type of natural bait on fish catches and hooking location in a mixed-species marine recreational fishery, with implications for management. Fisheries Research 97: 270–277.

[49] Bailey DM, Priede IG (2002) Predicting fish behaviour in response to abyssal food falls. Marine Biology 141: 831–840.

[50] Stoner AW, Ottmar ML, Hurst TP (2006) Temperature affects activity and feeding motivation in Pacific halibut: implications for bait-dependent fishing. Fisheries Research 81: 202–209.

[51] Collins MA, Yau C, Guilfoyle F, Bagley PM, Everson I, et al (2002) Assessment of stone crab (Lithodidae) density on the South Georgia slope using baited video cameras. ICES Journal of Marine Science 59: 370–379.

[52] Westerberg H, Westerberg K (2011) Properties of odour plumes from natural baits. Fisheries Research 110(3): 459–464.

[53] Meffe GK, Sheldon AL (1988) The influence of habitat structure on fish assemblage composition in southeastern blackwater streams. The American Midland Naturalist 120: 225–240.

[54] Charbonnel E, Serre C, Ruitton S, Harmelin JG, Jensen A (2002) Effects of increased habitat complexity on fish assemblages associated with large artificial reef units (French Mediterranean coast). ICES Journal of Marine Science 59: 208–213.

[55] Jaureguizer AJ, Menni R, Bremec C, Mianzan H, Lasta C (2003) Fish assemblage and environmental patterns in the Rio de la Plata estuary. Estuarine, Coastal and Shelf Science 56: 921–933.

[56] Anderson MJ, Millar RB (2004) Spatial variation and effects of habitat on temperate reef fish assemblages in northeastern New Zealand. Journal of Experimental Marine Biology and Ecology 305: 191–221.

[57] Pusey BJ, Kennard MJ, Arthington AH (2000) Discharge variability and the development of predictive models relating stream fish assemblage structure to habitat in northeastern Australia. Ecology of Freshwater Fish 9: 30–50.

[58] Moranta J, Palmer M, Morey G, Ruiz A, Morales-Nin B (2006) Multi-scale spatial variability in fish assemblages associated with *Posidonia oceanica* meadows in the Western Mediterranean Sea. Estuarine, Coastal and Shelf Science 68: 579–559.

[59] Spencer ML, Stoner AW, Ryer CH, Munk JE (2005) A towed camera sled for estimaing abundance of juvenile flatfishes and habitat characteristics: Comparison with beam trawls and divers. Estuarine, Coastal and Shelf Science 64: 497–503.

[60] Kenyon JC, Brainard RE, Hoeke RK, Parrish FA, Wilkinson CB (2006) Towed-diver surveys, a method for mesoscale spatial assessment of benthic reef habitat: A case study at Midway Atoll in the Hawaiian Archipelago. Coastal Management 34: 339–349.

[61] Kenyon JC, Brainard RE, Hoeke RK, Parrish FA, Wilkinson CB (2006) Towed-diver surveys, a method for mesoscale spatial assessment of benthic reef habitat: A case study at Midway Atoll in the Hawaiian Archipelago. Coastal Management 34: 339–349.

[62] Boersma PD, Parrish JK (1999) Limiting abuse: marine protected areas, a limited solution. Ecological Economics 31: 287–304.

[63] Nardi K, Jones GP, Moran MJ, Cheng YW (2004) Contrasting effects of marine protected areas on the abundance of two exploited reef fishes at the sub-tropical Houtman Abrolhos Islands, Western Australia. Environmental Conservation 31: 160–168.

[64] Pearce A, Lenanton R, Jackson G, Moore J, Feng M, et al (2011) The "marine heat wave" off Western Australia during the summer of 2010/11. Perth: Government of Western Australia, Department of Fisheries.

[65] Burdwick D, Brown V, Asher J, Gawel M, Goldman L, et al (2008) The State of Coral Reef Ecosystems of Guam. Center for Coastal Monitoring and Assessment, Maryland, United States.

[66] Brooks EJ, Sloman KA, Sims DW, Danylchuk AJ (2001) Validating the use of baited remote underwater video surveys for assessing the diversity, distribution and abundance of sharks in the Bahamas. Endangered Species Research 13: 231–243.

[67] Tacon AGJ, Metian M (2008) Global overview on the use of fish meal and fish oil in industrially compounded aquafeeds: Trends and future prospects. Aquaculture 285: 146–158.

[68] Gaughan DJ (2002) Disease-translocation across geographic boundaries must be recognized as a risk even in the absence of disease identification: the case with Australian *Sardinops* . Reviews in Fish Biology and Fisheries 11: 113–123.

[69] Hasson KW, Fan Y, Reisinger T, Venuti J, Varner PW (2006) White-spot syndrome virus (WSSV) introduction into the Gulf of Mexico and Texas freshwater systems through imported, frozen bait-shrimp. Diseases of Aquatic Organisms 71: 91–100. 16956056 10.3354/dao071091

[70] RSPCA. Cat Food Made Safer. 2011.

[71] Watson DL, Harvey ES (2009) Influence of the Leeuwin Current on the distribution of fishes and the composition of fish assemblages. Journal of the Royal Society of Western Australia 92: 147–154.

[72] Watson DL, Harvey ES (2007) Behaviour of temperate and sub-tropical reef fishes towards a stationary SCUBA diver. Marine and Freshwater Behaviour and Physiology 40: 85–103.

[73] Watson DL (2006) Use of underwater stereo-video to measure fish assemblage structure, spatial distribution of fishes and change in assemblages with protection from fishing. Dissertation, The University of Western Australia.

[74] SeaGIS. 2011. Camera calibration and bundle adjustment.

[75] Harvey ES, Shortis M (1996) A system for stereo-video measurement of sub-tidal organisms. Marine Technology Society Journal 29: 10–22.

[76] SeaGIS. 2011. Three-dimensional stereo measurement in visual sampling of fish population*s*.

[77] Cappo M, Harvey E, Malcolm H, Speare P (2003) Potential of video techniques to monitor diversity, abundance and size of fish in studies of Marine Protected Areas. In: Beumer JP, Grant A, Smith DC, editors. Aquatic Protected Areas - what works best and how do we know? Cairns, Australia, August 2002. p455–464: World Congress on Aquatic Protected Areas proceedings..

[78] Anderson MJ (2001) A new method for non-parametric multivariate analysis of variance. Austral Ecology 26: 32–46.

[79] Clarke KR, Warwick RM (2001) Change in Marine Communities: An approach to statistical analysis and interpretation. 2nd edition, Plymouth Marine Laboratory, United Kingdom: PRIMER-E Ltd.

[80] Zar JH (1999) Biostatistical Analysis. 4th Edition, Prentice Hall, New Jersey.

[81] Clarke KR, Somerfield PJ, Chapman MG (2006) On resemblance measures for ecological studies, including taxonomic dissimilarities and a zero-adjusted Bray-Curtis coefficient for denuded assemblages. Journal of Experimental Marine Biology and Ecology 330: 55–80.

[82] Anderson MJ (2006) Distance-based tests for homogeneity of multivariate dispersions. Biometrics 62: 245–253. 16542252 10.1111/j.1541-0420.2005.00440.x

[83] Anderson MJ, Willis TJ (2003) Canonical analysis of principal coordinates: a useful method of constrained ordination for ecology. Ecology 84: 511–525.

[84] Anderson MJ, Robinson J (2003) Generalized discriminant analysis based on distances. Australian & New Zealand Journal of Statistics 45: 301–318.

